# Cognitive performance recovery following marijuana cessation in NCAA division I athletes

**DOI:** 10.3389/fspor.2026.1821656

**Published:** 2026-06-02

**Authors:** Brandon A. Ally, Paul A. Mentele, Scott A. Wylie

**Affiliations:** 1 Department of Neurosurgery, University of Louisville, Louisville, KY, United States; 2 Medicine Institute, UofL Health, University of Louisville, Louisville, KY, United States

**Keywords:** cannabis, cognition, decision speed, inhibition, NCAA football, performance, S2 cognition, THC

## Abstract

**Background:**

Cannabis use is increasingly common among collegiate athletes. While acute tetrahydrocannabinol (THC) effects are well documented, the chronic impact on game-speed cognition, defined as the ability to perceive, decide, and act within sub-second windows, remains poorly quantified. The current study examined whether chronic THC use degrades football specific game-speed cognition and reaction times.

**Methods:**

Eighteen NCAA Division I football players who tested positive for THC completed the S2 Cognition Football Battery at baseline and again after 12 months of verified abstinence. The battery yields nine primary cognitive processes (Perception Speed, Search Efficiency, Tracking Capacity, Visual Learning, Instinctive Learning, Decision Complexity, Impulse Control, Distraction Control, Improvisation) and an overall S2 Composite, plus two secondary summary measures; reaction speed and decision accuracy. Paired *t*-tests and linear mixed-effects models (controlling for reaction speed) assessed change.

**Results:**

At baseline, chronic users showed approximately 20% slower reaction speed relative to their own post-abstinence values [t(17) = −5.84, *p* < .001], consistent with an ∼80–100 ms delay in decision execution. Significant improvements were observed in Decision Complexity, Distraction Control, Impulse Control, Multiple-Object Tracking, Speeded Target Detection, Improvisation, and the S2 Composite (all *p* < .002, Holm Method-corrected). Effects persisted after controlling for reaction speed, indicating domain-specific recovery beyond global speeding.

**Conclusion:**

Chronic THC use imposes a measurable performance cost by slowing speeded decision-making and degrading attention and control processes that support play execution; these effects are partially reversible with abstinence. Findings provide an objective, performance-based framework for athlete education.

## Introduction

Cannabis availability and use have risen with legalization across the United States, raising important questions about how tetrahydrocannabinol (THC) exposure affects competitive performance in collegiate sport. While public discussion often centers on policy and detection thresholds, the scientifically salient question for athletes is whether THC compromises game-speed cognition or decision-making. For current purposes, we define game-speed cognition as the sub-second perception, decision, and action processing that governs performance under pressure. Converging experimental and neuroimaging data indicate that THC impairs the speed and coordination of many of these processes, yet few studies have quantified sport-relevant costs in elite athletes or assessed recovery with sustained abstinence.

Acute and subacute THC impairment is well documented. Studies show acute THC slows reaction speed, degrades sustained/selective attention, and reduces visuomotor precision across administration routes (i.e., smoking, edibles, etc.) and dosage (1–4). THC also impairs longitudinal control during complex tasks (e.g., instrumented driving), underscoring higher-order sensorimotor liabilities under dual-task demands (5). Importantly, subjective intoxication and measurable neurocognitive impairment do not necessarily resolve on identical time courses. Controlled studies indicate that performance can remain impaired after the subjective “high” has begun to dissipate and when circulating peripheral THC concentrations are relatively modest (1, 4). Accordingly, peripheral THC concentrations should be interpreted cautiously, as they may not fully capture the persistence of THC-related cognitive and psychomotor effects (6).

With repeated exposure, broader disturbances in executive function, attention, and decision-making emerge (7–9). Neuroimaging studies link chronic use to altered recruitment and coupling within prefrontal and striatal systems that support inhibitory control and conflict monitoring (10), as well as cerebellar changes with implications for the temporal precision of visuomotor behavior (11, 12). Behaviorally, chronic users show slower, less accurate performance on cognitive-control paradigms (e.g., Simon/Flanker), reflecting impaired resolution of response conflict and reduced flexibility (8, 13, 14).

Complementary literature highlights visual and oculomotor liabilities: increased saccadic latency, reduced tracking accuracy, and impaired visual scanning efficiency with THC exposure (15), and poorer adaptation in visuomotor rotation (16). These findings are critical to sports because field vision depends on spatial awareness, multi-target updating, and rapid extraction of task relevant cues from clutter. These time limited processes likely degrade with slowed perception and unstable oculomotor control.

Mechanistically, THC engages CB1 receptors across cortico-striato-cerebellar networks, perturbing excitation/inhibition balance, timing precision, and synaptic efficiency in circuits that implement fast perception—action coupling and inhibitory gating (11, 17). Developmental and clinical work shows that repeated exposure can reshape transcriptional trajectories and connectivity, particularly in frontal systems (18, 19), and increase motor impulsivity (20). Together, these data predict impaired speeded cognition decision making and compromised executive control under game speed constraints.

Despite this literature, there is limited evidence directly quantifying the magnitude of THC-related slowing in high-level athletes on sport-relevant cognitive tasks, and whether those costs reverse with abstinence. The S2 Cognition Football Battery provides a validated, sport-specific framework used by NCAA and professional programs to assess nine primary cognitive processes and an overall composite, alongside secondary measures indexing the reaction speed and accuracy of decisions when responses must occur in <500 ms. In the present study, we tested NCAA Division I athletes at baseline (chronic use) and after 12 months of abstinence to quantify (a) cognitive specific changes most directly related to football performance and (b) the degree of slowing in speeded decision making. We predicted slower, less efficient decision execution during chronic use and measurable, domain-specific recovery following abstinence (2, 3, 7, 11). Specifically, based on previous literature, we predicted that during periods of chronic THC usage, players would have significantly decreased performance on motor control and execution tasks (i.e., Decision Complexity, Impulse Control, Distraction Control, and Improvisation) compared to their period of abstinence. Secondarily, we predicted that during periods of chronic THC usage, overall reaction speed would be significantly slower than during periods of abstinence.

## Materials and methods

### Participants

Participants were 22 male NCAA Division I football players (mean age = 19.9 ± 1.6 years) who tested positive for THC during preseason onboarding (≥150 ng/mL, consistent with NCAA/WADA-aligned screening standards). Each one of the players also subjectively acknowledged being chronic smokers and endorsed “daily or near daily use”. None of these players were in a concussion protocol or reported a head injury restricting activity within the prior three months. No athlete endorsed having used THC in the morning of the evaluation. All had normal or corrected-to-normal vision. Further, all participants were encouraged to engage in testing in the state in which they play the game (e.g., caffeine, ADHD meds, etc.). Eighteen (18) athletes (mean age = 19.6 ± 1.5) maintained program verified abstinence for 12 months between test sessions and were included in analyses.

### Procedures

Testing occurred at two time points separated by 363 days in a controlled laboratory setting. The S2 Cognition Football Battery was administered on calibrated computers with millisecond timing precision using a response interface providing 2–3 ms resolution (Cedrus Button Box). Tasks require rapid perception and response execution under time pressure to emulate competitive demands. Scores are standardized to a large collegiate-athlete normative dataset (*n* = 4,600), yielding z-scores for each primary process and the overall composite.

### S2 cognitive constructs

The battery quantifies nine primary cognitive processes relevant to football: Perception Speed [speed of processing visual details within central vision; modified version of (21)]; Search Efficiency [efficiency of scanning to locate a target in visual clutter; modified version of (22)]; Tracking Capacity [multiple object tracking; modified version of (23)]; Visual Learning [visual short term working memory; modified version of (24)]; Instinctive Learning (probabilistic learning; modified version of (25)]; Decision Complexity [speed/efficiency of selecting a correct option under multiple rules conditions; methods previously reported in (26)]; Impulse Control [rapid inhibition of motor response under time pressure; modified Simon task, methods previously reported in (27)]; Distraction Control [shielding motor execution from external distractions; modified Flanker Task, methods previously reported in (28)]; and Improvisation [speed and flexibility of counter-reactions when situations change unexpectedly; methods previously reported in (26)]. The Overall S2 Composite measure is the average z-score across all nine cognitive tasks. The two secondary measures, SPEED (how fast decisions are executed when responses must occur in <500 ms) and ACC (how precisely those decisions are executed) are also z-scores compared to the normative database. The S2 Cognition Football Battery has been field-validated and is used by multiple NCAA programs and NFL teams to quantify game-speed cognition.

### Statistical analysis

Z-scores for primary processes and the S2 Composite were analyzed with paired-sample *t*-tests comparing pre- and post-abstinence performance. All tests were evaluated at α = 0.05 using the Holm-Bonferroni method to control for multiple comparisons. Effect sizes were expressed as Cohen's d, and counts of individual improvements vs. declines were tabulated. To determine whether changes reflected only global speeding, linear mixed-effects models were fit for each cognitive variable with a random intercept for participant and SPEED as a fixed-effect covariate. Analyses were conducted in Python 3.11.5 using statsmodels 0.14.4 and SciPy 1.15.2.

## Results

**Figure 1:**
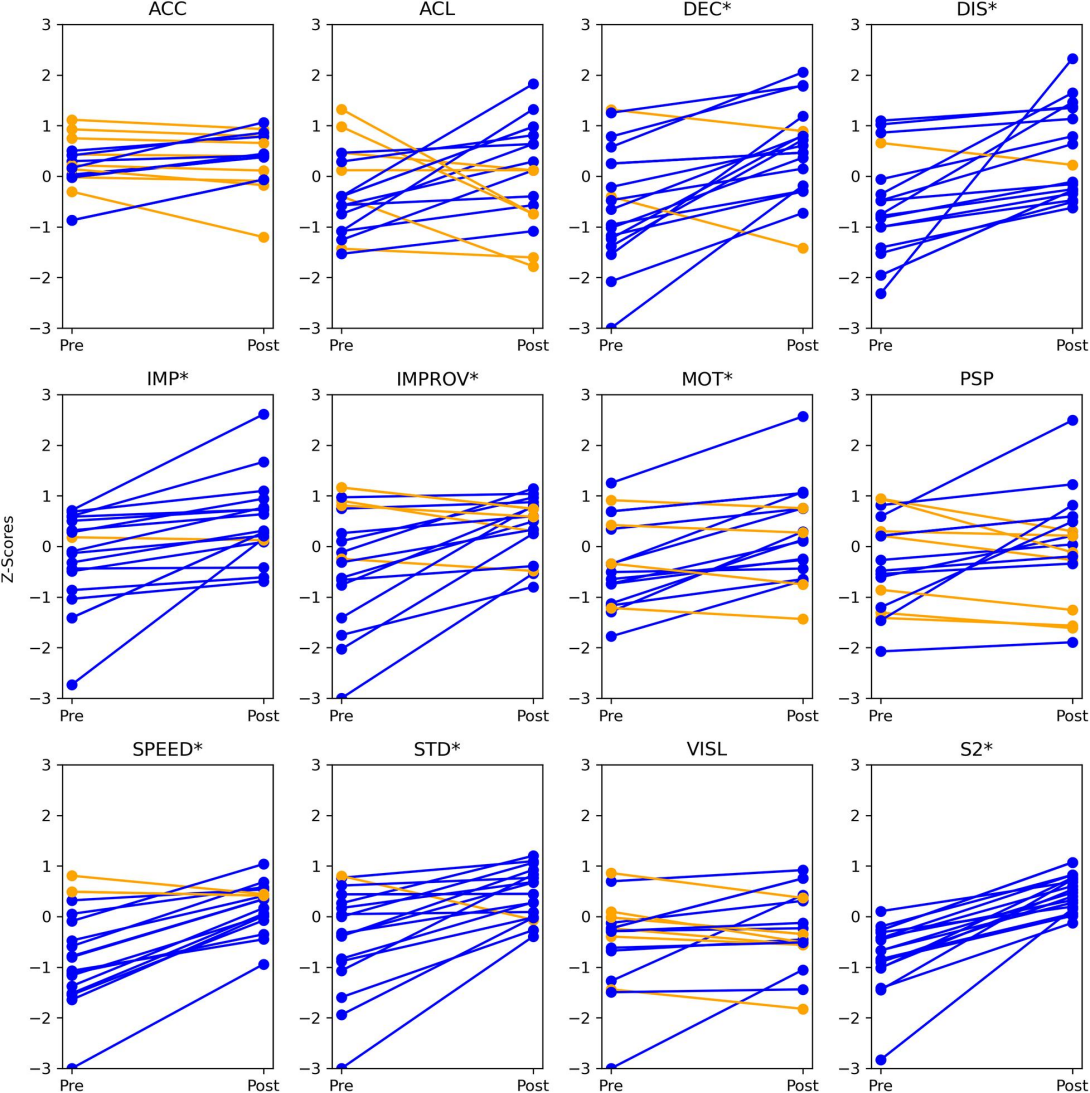
Spaghetti plots showing within-athlete changes in standardized cognitive performance from pre- to post-abstinence testing across 12 S2 measures: ACC, ACL, DEC*, DIS*, IMP*, IMPROV*, MOT*, PSP, SPEED*, STD*, VISL, and S2 Overall*. Each line represents one athlete's paired Z-scores across time. Blue lines indicate improvement at post-testing, whereas orange lines indicate no change or worsening. Measures marked with an asterisk (*) were statistically significant after multiple-comparison correction..

Significant improvements were observed in Decision Complexity [t(17) = −4.44, *p* < .001], Distraction Control [t(17) = −4.04, *p* < .001], Impulse Control [t(17) = −4.19, *p* < .001], Multiple-Object Tracking [t(17) = −3.92, *p* = .001], Speeded Target Detection [t(17) = −4.20, *p* < .001], and the S2 Composite [t(17) = −9.07, *p* < .001] (Table 1). Accuracy (ACC), Instinctive Learning, Improvisation, Visual Learning, and Perception Speed showed numerical gains that did not survive Bonferroni correction. Effect sizes were large for significant domains (d ≈ 0.95–2.20). Post-hoc mixed-effects models controlling for reaction speed confirmed that improvements in Decision Complexity [β = 1.049, SE = 0.236, 95% CI (0.586–1.511), *p* < 0.001], Distraction Control [β = 1.066, SE = 0.264, 95% CI (0.548–1.511), *p* < 0.001], Impulse Control [β = 0.776, SE = 0.185, 95% CI (0.413–1.139), *p* < 0.001], Multiple-Object Tracking [β = 0.570, SE = 0.145, 95% CI (0.285–0.855), *p* < 0.001], Speeded Target Detection [β = 0.827, SE = 0.197, 95% CI (0.441–1.213), *p* < 0.001], Improvisation [β = 0.680, SE = 0.230, 95% CI (0.229–1.130), *p* = 0.003], and the S2 Composite [β = 1.266, SE = 0.140, 95% CI (0.992–1.540), *p* < 0.001] remained statistically significant, indicating domain-specific recovery beyond global speeding (Table 2). Individual pre- to post-abstinence trajectories for each cognitive measure are shown in Figure 1, with the largest and most consistent upward shifts evident for DEC, DIS, IMP, MOT, SPEED, STD, and S2 Overall. Reaction speed across cognitive tasks was approximately 20% slower when chronically using THC compared to post-abstinence reaction speed [t(17) = −5.84, *p* < .001], corresponding to an estimated ∼80–100 ms delay in decision execution within sub-second windows.

**Table 1:** Descriptive statistics for cognitive measures pre- and post-THC cessation.

Measure	Pre-mean (SD)	Post-mean (SD)	Improved (worsened)	Effect size (d)
S2 Overall*	−0.773 (0.669)	0.493 (0.356)	18 (0)	2.200
SPEED*	−0.722 (0.961)	0.202 (0.461)	15 (3)	1.416
DEC*	−0.539 (1.150)	0.509 (0.897)	16 (2)	1.077
STD*	−0.382 (1.023)	0.445 (0.488)	17 (1)	1.019
IMP*	−0.281 (0.925)	0.495 (0.794)	17 (1)	1.017
DIS*	−0.627 (1.034)	0.439 (0.911)	17 (1)	0.979
MOT*	−0.329 (0.867)	0.241 (0.928)	14 (4)	0.951
IMPROV	−0.267 (1.194)	0.413 (0.589)	13 (5)	0.717
ACC	0.160 (0.521)	0.332 (0.536)	10 (8)	0.391
PSP	−0.334 (0.918)	−0.016 (1.084)	11 (7)	0.370
VISL	−0.482 (0.885)	−0.246 (0.745)	11 (7)	0.332
ACL	−0.347 (0.814)	0.049 (1.002)	12 (6)	0.328

Mean (SD). Improved (Worsened) shows number of participants by direction of change.

* Indicates significance after Bonferroni correction (α = .002).

**Table 2:** Linear mixed-effects model results controlling for SPEED.

Measure	Coefficient (β)	SE	*p*-value	95% CI
DEC*	1.049	0.236	<0.001	0.586–1.511
STD*	0.827	0.197	<0.001	0.441–1.213
IMP*	0.776	0.185	<0.001	0.413–1.139
DIS*	1.066	0.264	<0.001	0.548–1.583
MOT*	0.570	0.145	<0.001	0.285–0.855
IMPROV*	0.680	0.230	0.003	0.229–1.130
ACC	0.172	0.107	0.107	−0.037–0.382
PSP	0.318	0.208	0.127	−0.090–0.726
VISL	0.236	0.172	0.170	−0.101–0.573
ACL	0.397	0.293	0.176	−0.178–0.972

Regression coefficients (β), standard errors (SE), *p*-values, and 95% confidence intervals.

* Indicates significance using the Holm Method.

To better contextualize the possibility that the observed improvements reflect maturation, football-specific development, or repeated exposure to the assessment, we include reference data from an independent football comparison sample assessed approximately one year apart. In that sample (*n* = 146; mean interval = 11.29 months), overall S2 performance was highly stable across time (r = .897), with only modest average year-over-year change (+0.14 change in z-score), and individual domains demonstrated moderate-to-strong longitudinal stability (Pearson r = .70–.81; ICC = .70–.80).

## Discussion

In line with our apriori hypotheses, significant improvements after abstinence were seen in Decision Complexity (DEC), Distraction Control (DIS), Impulse Control (IMP), Multiple-Object Tracking (MOT), Speeded Target Detection (STD), and the overall S2 composite. Mixed-effects models indicated that cognitive domain gains persisted after controlling for reaction time, suggesting that abstinence supports recovery not only of global decision speed, but also of process specific cognitive control functions. Additionally, athletes exhibited approximately 20% slower reaction speed when chronically using compared to their abstinent state (i.e., 80–100 ms delay in decision execution within sub-second windows). This 80–100 ms slowing observed here should not be interpreted in the abstract, but in relation to the timing of the game. In NFL pass-rush data, edge defenders commonly reach the line of scrimmage in well under one second, with the fastest averaging about 0.73–0.76 s and even slower rushers generally below 1.10 s (29). This includes not only the cognitive reaction time to the snap, but also the physical act of getting to the line of scrimmage. An offensive player who is even modestly slower in decision execution is operating with less time to identify, select, and initiate the correct response. In that sense, the present results suggest a plausible football-specific performance disadvantage associated with chronic THC use, although direct on-field outcomes were not measured in this sample. Supporting this interpretation, Bowman et al. (30) reported that offensive linemen with slower reaction times exhibited higher rates of false-start penalties. However, because Bowman et al. did not report raw reaction-time values and the assessment was administered on an iPad, the precision and accuracy of those timing estimates remain difficult to evaluate.

The findings of the current study align with controlled studies showing that THC slows reaction speed and impairs attentional control and visuomotor precision under cognitive load (1–4). The current results show that chronic THC usage, even when not actively “high”, impacts the speed to select from multiple stimulus-response options (DEC), shield out distractions from impacting motor performance (DIS), inhibit premature responses (IMP), track multiple moving objects (MOT), and locate a target in visual chaos (STD). These findings are consistent with established THC related impairments in executive control and attention (8–10) and visual-oculomotor processing (15, 16). Supporting this, neuroimaging studies have found reorganized visual-attention/cerebellar (11) and altered frontostriatal recruitment during tasks of conflict resolution networks in chronic users (13). The within-athlete abstinence design extends this literature by demonstrating substantial recovery over 12 months, supporting evidence that psychomotor and executive deficits can normalize with sustained cessation (31), and by quantifying change in sport-specific metrics used in elite programs (12).

Mechanistically, CB1-receptor activation perturbs timing precision and synaptic efficiency in prefrontal–striatal–cerebellar networks that support rapid response selection and inhibition (11, 12, 17). Chronic exposure to THC is associated with altered white-matter microstructure and network recruitment (10, 19), slower oculomotor control (15), and impaired motor adaptation (16). Abstinence-related recovery likely reflects partial normalization (e.g., receptor resensitization; restoration of excitation-inhibition balance), consistent with observed functional improvement following sustained cessation (31). Preclinical data showing transcriptional and dendritic remodeling with THC exposure (18) provide plausible substrates for both impairment and recovery trajectories.

Functionally, the affected domains have direct football implications. Slower decision complexity elongates time to commit to action when multiple rules or options are available (progression reads, read-option routes for receivers, gap fit choices). Weaker distraction control increases susceptibility to motion, play-action, hands in face, hearing footsteps, and exhibiting poor eye discipline for defenders. Reduced impulse control elevates premature reactions (e.g., biting on double moves, grabbing jerseys, throwing prematurely into traffic). Lower multiple object tracking reduces field vision, limiting simultaneous awareness of crossers, pullers, and backfield flow. Poorer speeded target detection delays pickup of task-relevant cues (safety rotation, nickel blitz, RB release). Together, these functions determine how efficiently an athlete converts perception into decisive, accurate action under time pressure. Evidence from the current study highlights the significant detriment to play speed and decision when chronically using THC product. Further, recent literature shows that reduced performance on tasks of speeded cognition puts athletes at higher risk for soft tissue injury (32). Chronic THC usage may indirectly lead to higher injury rates in college and professional football players (33).

In summary, chronic THC use slows speeded decision-making and degrades executive control processes essential for football performance, while one year of abstinence yields broad cognitive recovery that extends beyond global speeding. By translating neuroscience into sport-specific metrics, these findings furnish a rigorous, athlete centered basis for informed decisions about THC use.

## Limitations

This study should be interpreted in light of several limitations. First, it originated as an applied athlete-support evaluation within a university football program rather than a prospectively designed research protocol. As a result, several baseline variables that would strengthen causal inference were not systematically collected, including detailed THC exposure history (e.g., exact frequency, dose, potency, concurrent edible use), route(s) of administration beyond self-reported smoking, and standardized quantification of other substances such as caffeine or nicotine. Second, the study did not include a matched non-THC control group or randomized assessment order, so the observed improvements cannot be attributed solely to abstinence. Although an independent football comparison sample assessed approximately one year apart showed only modest average change in overall S2 performance (about +0.14 SD) with high longitudinal stability, these data do not eliminate the possibility that maturation, football-specific training, or retest effects contributed to improvement in the present cohort. Third, direct on-field football outcomes were not measured in this sample. Accordingly, the practical implications discussed here should be interpreted as translational rather than as direct evidence that the observed cognitive differences produced measurable changes in game performance. Finally, the sample was limited to male NCAA Division I football players, which constrains generalizability to other sports, female athletes, and non-athlete populations. The current manuscript's design and sample description reflect this applied within-athlete, two-timepoint framework.

## Data Availability

The raw data supporting the conclusions of this article will be made available by the authors, without undue reservation.
